# What constitutes optimal care coordination for primary brain tumors and how do we assess it in Australia and Aotearoa New Zealand? A Delphi consensus study

**DOI:** 10.1093/nop/npaf082

**Published:** 2025-08-04

**Authors:** Megan S Jeon, Sharon He, Joanne Shaw, Eng-Siew Koh, Brian Kelly, Mark B Pinkham, Dianne M Legge, Georgia K B Halkett, Raymond J Chan, Tamara Ownsworth, Ursula M Sansom-Daly, Marina Kastelan, Haryana M Dhillon, the BRAINS Investigator Group, Megan S Jeon, Sharon He, Joanne Shaw, Eng-Siew Koh, Brian Kelly, Mark B Pinkham, Dianne M Legge, Georgia K B Halkett, Raymond J Chan, Tamara Ownsworth, Ursula M Sansom-Daly, Marina Kastelan, Haryana M Dhillon

**Affiliations:** Faculty of Science, Psycho-oncology Co-operative Research Group (PoCoG), School of Psychology, The University of Sydney, Sydney, New South Wales, Australia; Faculty of Science, Psycho-oncology Co-operative Research Group (PoCoG), School of Psychology, The University of Sydney, Sydney, New South Wales, Australia; Faculty of Science, Psycho-oncology Co-operative Research Group (PoCoG), School of Psychology, The University of Sydney, Sydney, New South Wales, Australia; South West Sydney Clinical School, University of New South Wales, Sydney, New South Wales, Australia; Liverpool Cancer Centre, Liverpool Hospital, South Western Sydney Local Health District, Sydney, New South Wales, Australia; School of Medicine and Public Health, University of Newcastle (UON), Callaghan, New South Wales, Australia; Faculty of Medicine, University of Queensland, St Lucia, Queensland, Australia; Department of Radiation Oncology, Princess Alexandra Hospital, Woolloongabba, Queensland, Australia; Curtin School of Nursing, Curtin University, Perth, Western Australia, Australia; Oliva Newton-John Cancer and Wellness Centre, Austin Hospital, Heidelberg, Victoria, Australia; Curtin School of Nursing, Curtin University, Perth, Western Australia, Australia; Faculty of Health Sciences, Caring Futures Institute, College of Nursing and Health Sciences, Flinders University, Bedford, Western Australia, Australia; The Hopkins Centre, School of Applied Psychology, Griffith University, Mt Gravatt, Queensland, Australia; Sydney Youth Cancer Service, Nelune Comprehensive Cancer Centre, Prince of Wales Hospital, Randwick, New South Wales, Australia; Kids Cancer Centre, Sydney Children’s Hospital, Randwick, New South Wales, Australia; Behavioural Sciences Unit, Discipline of Paediatrics and Child Health, School of Clinical Medicine, UNSW Medicine & Health, University of New South Wales, Kensington, New South Wales, Australia; The Brain Cancer Group, North Shore Private Hospital, St Leonards, New South Wales, Australia; Faculty of Science, Psycho-oncology Co-operative Research Group (PoCoG), School of Psychology, The University of Sydney, Sydney, New South Wales, Australia

**Keywords:** brain cancer, care coordination, Delphi, expert consensus, framework

## Abstract

**Background:**

People with primary brain tumors (PBT) and their carers experience diverse issues and functional impairments, which can form barriers to accessing tailored health care. Care coordination (CC) addresses this dual challenge and is critical to achieving high-quality care for PBT. We aimed to develop a consensus framework and assessable quality indicators for optimal CC for PBT.

**Methods:**

A 2-phase, modified Delphi process was conducted. In Phase 1, a preliminary framework of 4 domains and 140 items was identified from a scoping review and expert stakeholder advisory group discussion (*n* = 14). In Phase 2, multidisciplinary panel members (*n* = 40) with expertise in clinical management and support for PBT indicated level of agreement (consensus criteria: ≥80% agreement and a median score of ≥4) on proposed items using a 5-point Likert scale. The expert stakeholder advisory group finalized components and indicators based on panel consensus findings.

**Results:**

Consensus was achieved for 97/140 items across four domains (definition, objective, components, and indicators), and a further 33 items approached consensus following 2 survey rounds. Panelist ratings on quality indicators for CC varied, especially for items related to indicators of healthcare system governance (range 48%–79% agreement). The expert stakeholder advisory group finalized the inclusion of 6 additional items based on feedback from panelists, producing a final list of 136 items.

**Conclusions:**

We defined a novel framework of CC specific to PBT, which presents a consensus-based definition and objectives for CC and comprehensive lists of components and quality indicators. The framework provides a useful template for developing models of CC.

**Key Points**

- Experts and stakeholders have defined care coordination and its objectives for primary brain tumors.
- A novel framework identifies components and indicators of optimal care coordination.

**Importance of the Study**

Primary brain tumors (PBT) constitute diverse and rare tumor subtypes, which pose a wide range and unique set of challenges to people with PBT and their families. PBT treatment and survivorship present complexity and challenges for patients and carers to access multidisciplinary care and appropriate support. While care coordination is critical to achieving high-quality care for PBT, a framework of optimal care coordination to guide clinical practice is lacking internationally. We present a novel framework of care coordination for people with PBT derived through a multistage Delphi consensus process. This framework includes a total of 136 items, encompassing the definition and objectives of care coordination, as well as a comprehensive list of components and indicators to guide the development and evaluation of an optimal care coordination model in neuro-oncology. Further research investigating the clinical utility of the proposed framework, including development, implementation, and evaluation of models of care coordination, is warranted.

Individuals living with primary brain tumors (PBT) and their family carers experience a wide range and unique set of challenges, including neurological, physical, cognitive, and psychological symptoms and treatment side effects, throughout their disease trajectory, resulting in high levels of distress, morbidity, unmet needs, poor health-related quality of life, and psychosocial impacts.^1–5^ PBT treatment and survivorship present complexity and challenges for people with PBT and carers to access continuous, integrated, and timely care while navigating multidisciplinary specialist healthcare services (eg, neurosurgery, medical and radiation oncology, allied health, palliative care), as well as nonclinical support services (eg, financial and legal) across hospital sites and community care settings. Coordination of treatment and supportive care is highly valued in PBT care, bringing together different components of needed care in a timely and cohesive manner for individuals and families according to their medical and psychosocial needs.^6^ Care coordination (CC) has been proposed as one of the principles underpinning high-quality, patient-centered care for people with cancer, including high-grade glioma, as per the Optimal Care Pathway for High-Grade Glioma.^7^

A systematic review and meta-analysis of cancer CC interventions (*n* = 52 studies) in North America suggests cancer CC improved care processes, appropriate healthcare utilization, and patient-level outcomes (eg, quality of life) with small–medium effects.^8^ The most common CC interventions identified were patient navigation, followed by home telehealth and nurse case management. This reflects a predominant approach in cancer CC focusing on health systems providing cost-effective care (avoiding duplication) and reducing barriers to timely access to appropriate health services for individuals and families affected by cancer.^9^ However, there was considerable heterogeneity in outcomes measured and intervention protocols included. The quality of reporting of studies on CC activities varied, and it was unclear if people with PBT were represented, as most included studies reported mixed cancers, breast, or lung cancer populations. Interventions targeting varying care levels and across the cancer continuum, especially survivorship, were lacking.

Similarly, in Australia and Aotearoa New Zealand (AANZ), 2 neighboring countries with similar healthcare systems, no single model of CC in neuro-oncology proposes features widely applicable to different healthcare settings in AANZ. The current approach to CC relies on a small number of designated brain cancer care coordinators, whose roles and positions within the multidisciplinary care team have defined a model of CC in individual institutions.^10,11^ However, access to designated care coordinators, referral pathways, supportive and psychosocial care, and resources varies across geographical locations or health settings where people with PBT and their carers live and receive treatment.^12^

There is a lack of consensus regarding the framework of CC as a *process or function* within a brain tumor care model, beyond a set of duties or activities for a care coordinator. Furthermore, empirical data to guide the development of a framework of optimal CC for PBT is lacking.^13^ In our recent scoping review, we identified variable concepts and objectives of CC, with a range of components and outcomes of CC for people with PBT.^13^ While CC in the brain cancer context has similarly focused on healthcare system navigation, improved access to medical care, and timely brain cancer diagnosis and treatment within tertiary care centers, most components of CC did not reflect factors specific to neuro-oncology care.^13^ For example, neurological symptoms, functional impairment, cognitive issues, and varying distress levels are additional CC concerns for people with PBT,^14^ as these symptoms and treatment effects of neurosurgery and/or subsequent treatment are highly debilitating, impairing the ability of individuals with both benign and malignant PBT to navigate health care and maintain quality of life during survivorship when unaddressed as part of CC.^15^ Involving carers in PBT care was an important facilitator and unique consideration given their level of support provision and involvement in care decision-making due to reduced functional capacity and mobility of the patients. However, no systematic attempt has been made to determine specific care requirements of PBT and adapt definitions and frameworks of CC to this population. In addition, similar challenges in defining indicators of effective CC have been found in the PBT context.^8,10^

Hence, we aimed to develop a consensus framework and assessable quality indicators for the delivery of optimal CC for PBT. Components and indicators of models of optimal CC can be systematically implemented and monitored to benchmark people’s experience with PBT care.^16^ From the scoping review, we devised an evidence-based preliminary framework of CC for people with PBT, also drawing from wider CC and general cancer research. In the current study, we aimed to gather opinions of experts and stakeholders caring/advocating for adults with PBT and their carers and achieve consensus regarding components of optimal CC and indicators of quality coordination of PBT care.

## Methods

### Design

We used a mixed-methods exploratory approach with a modified Delphi methodology. Given the lack of empirical evidence and limited clinical guidelines addressing CC for people with PBT, we sought expert opinions to develop a consensus concept of CC. The Delphi method is useful and appropriate as it addresses clinical questions or a lack of clarity in the concept “beyond the currently known or believed” guiding the implementation of pooled expert knowledge in clinical practice.^17^ The method used was informed by recent Delphi studies in clinical settings^18,19^ and follows the Guidance on Conducting and Reporting DElphi Studies (CREDES) developed for palliative care.^20^ The study was approved by the University of Sydney Human Ethics Research Committee [2023/493].

### Participants

#### Eligibility.—

We purposively recruited professionals and stakeholders with diverse perspectives and experiences of PBT as the multidisciplinary panel members, including (but not limited to) medical, nursing, psychology, and allied health professionals, consumer stakeholders and members of community organizations, and researchers experienced in working with adults (18 years or older) affected by PBT across AANZ. Participants were excluded if they were unable to complete online surveys in English; their clinical, research, support, or lived experience was solely with pediatric brain cancer; or they lived/worked in countries other than AANZ.

#### Recruitment of the expert stakeholder advisory group.—

We formed an expert stakeholder advisory group comprising healthcare professionals (HCPs), researchers, and consumers in neuro-oncology to provide clinical and contextual input throughout the Delphi process. Professionals (with >5 years of clinical and/or research experience in neuro-oncology) and consumer (individuals with PBT, caregivers, and community group representatives) stakeholders who were members of the Brain Cancer Rehabilitation Assessment Interventions for Survivorship Needs (BRAINs) Program were recruited via email invitation by the program manager. The expert stakeholder advisory group (*n* = 14) consisted of 8 professional stakeholders (4 healthcare professionals and 4 researchers) and 6 consumer stakeholders, with an average of 14 years of experience in brain tumor support or advocacy in Australia (Table 1).

**Table 1.** Characteristics of the Expert Stakeholder Advisory Group (*n* = 14)

	Professional (*n* = 8)		Consumer (*n* = 6)	
	Mean (range)		Mean (range)	
Age	49 (37–63)		49.8 (39–61)	
Years of clinical experience	21 (5–32)			
Years of experience in neuro-oncology care, research, or support/advocacy	17.25 (14–30)		9.4 (5–15)	
Years of experience in care coordination/case management/patient navigation, if applicable (*n* = 4)	19.75 (14–30)			
	*N*	%	*N*	%
Gender				
Female	5	63	6	100
Male	3	38		
Aboriginal or Torres Strait Islander Origin				
No	8	100	6	100
Language as child				
English	7	88	6	100
Other	1	13		
Language spoken at home				
English	7	88	6	100
Other	1	13		
Country of birth				
Australia	6	75	3	50
Other	2	25	2	33
Missing			1	17
Highest level of education				
Higher degree	7	88	2	33
Bachelor			2	33
High school			1	17
Missing	1	13	1	17
State of workplace				
New South Wales	4	50	1	17
Victoria	1	13	3	50
Other^a^	3	38	2	33
Location of work				
Metropolitan areas/major city	8	100	3	50
Regional/rural			3	50
Provision of services in regional or rural areas				
Yes	5	63	3	50
No	3	38	3	50
Brain tumor experience/expertise^b^				
High grade	8	100	3	50
Benign	4	50	2	
Lower grade	6	75	2	
Metastatic tumor	5	63		
Lived experience (consumer only)				
Yes			5 ^c^	83
No			1	17
Primary role in relation to brain tumor (professional only)				
Researcher	4	50		
Healthcare professional	4	50		
Professional discipline (professional only)				
Radiation oncologist	2	25		
Oncology nurse	1	13		
Radiation therapist	1	13		
Occupational therapist	1	13		
Cancer care coordinator	1	13		
Psychiatrist	1	13		
Psychologist	1	13		
Clinical setting (professional only)				
Tertiary cancer center/University	5	63		
District/local hospital—Inpatient	1	13		
Private hospital	1	13		
Private cancer treatment center—Outpatient	1	13		

Percentages are rounded to the nearest integers, and the sum may not equal 100%.

^a^Other states include Western Australia, Queensland, and South Australia.

^b^Multiple selection allowed.

^c^ *n* = 1, former carer.

#### Recruitment of the multidisciplinary expert panel members (study participants).—

Members of 5 AANZ interdisciplinary professional peak bodies and 2 community organizations were sent an email invitation from their organization with details about the study and a web link to the participant information statement and questionnaires. Implied consent to participate was provided with the submission of completed questionnaires. Organizations approached included Cooperative Trials Group for Neuro-Oncology (COGNO), Psycho-Oncology Cooperative Research Group (PoCoG), Oncology Social Work Australia New Zealand, Clinical Oncology Society of Australia (COSA) Neuro-oncology group, Primary Care Collaborative Cancer Clinical Trials Group (PC4), the Brain Cancer Group, and Peace of Mind Foundation. Participants were also recruited via the study investigators’ professional networks, study advertisements on social media platforms, and passive snowballing. Demographic and clinical practice or support information was obtained to confirm eligibility and relevant PBT experience and specialty.

### Delphi Process

The study was conducted in 2 phases, namely, item generation and consensus (Supplementary Figure 1).

#### Preparatory literature review.—

A scoping review of the literature was conducted in parallel with item generation.^13^ Key sources of information on care pathways for people with PBT and general cancer were also identified for potential relevance to, and features of, CC. An initial list of the concept, components, and indicators of CC for people with PBT (a preliminary framework) was developed to guide the design of Phase 1 focus group discussions (see Supplementary Materials).

#### Phase 1: item generation.—

Phase 1 was conducted from August to October 2023. The expert stakeholder advisory group was established to brainstorm, generate, and refine relevant items to address components and indicators of optimal CC for PBT. Professional stakeholders of the expert stakeholder advisory group met to discuss the preliminary framework of CC for PBT by videoconference to generate and organize items. Individual interviews and a focus group were held with consumer stakeholders of the expert stakeholder advisory group to obtain feedback and further input into the list of items. Investigators concealed the identity of the consumer stakeholders and provided anonymous written feedback to the professional stakeholders to reduce the potential risk of bias. Across 2 subsequent focus groups, the professional stakeholders further refined the items based on consumer feedback to present in the Delphi survey rounds in Phase 2. Items were organized by 4 domains of CC: **Definition**, **Objectives**, **Components**, and **Indicators** (for definition, see Supplementary Materials).

#### Phase 2: consensus.—

Phase 2 was conducted from October 2023 to April 2024 and aimed to gain consensus through a multiround iterative Delphi survey using online questionnaires via the Research Electronic Data Capture (REDCap) platform.^21^ In Round 1, participants were asked to complete a short demographic online survey and provide their primary email address before being directed to the first questionnaire. The questionnaire comprised items relevant to CC for PBT for participants to rate the level of agreement with or importance of each item, and free-text options for clarification of ratings or to suggest new items or alternative wording or phrases. In Round 2, items not reaching consensus in Round 1 were re-presented. Modifications to items were made and new items were introduced based on the results of the previous round, reflecting the iterative approach of Delphi methodology.^22^ Participants were invited by email to complete the Round 2 online questionnaire and share their feedback in free-text comments. Each item was presented with anonymous aggregate results of the previous round, including the frequency distribution for group ratings and a summary of free-text responses where applicable, in order to provide participants with the opportunity to reflect and reposition their opinions accordingly.^22^ The online questionnaire for each round took approximately 15–30 min to complete and was open for 4–6 weeks.

Consumer stakeholders of the expert stakeholder advisory group received aggregate results of the Round 2 survey and were asked to provide written feedback and advice to include or exclude items that did not reach consensus. A final focus group (Round 3) involving professional stakeholders of the expert stakeholder advisory group was held via videoconference, where they discussed these items and finalized consensus decisions (ie, retain, revise, or remove) based on the Round 2 results, consumer feedback, and investigators’ recommendations.

### Definition of Consensus

Items were grouped and presented within a domain of CC in online questionnaires. We used the modified UCLA RAND appropriateness method,^23^ using a 5-point Likert integer scale to promote clear differentiation between scale choices and reduce ambiguity and respondent burden from completing a questionnaire, as done in previous Delphi studies.^24^

Consensus for inclusion of an item was defined as ≥80% agreement and a median score of ≥4 on a 5-point Likert rating scale indicating the level of agreement (1, strongly disagree—5, strongly agree) with a statement or the level of importance (1, least important—5, most important) of a given component/indicator item for CC for people with PBT. Similarly, consensus for exclusion of an item was defined as ≥80% agreement and a median score of ≤2 on the same 5-point Likert rating scale. Items not meeting the consensus criteria were re-presented in subsequent survey rounds.

### Data Analysis

The audio recordings of interviews and focus groups with the expert stakeholder advisory group were used to generate a de-identified written summary of the group discussion. Online demographic and Delphi questionnaires were created on the REDCap platform.^21^ Descriptive and summary statistics were computed for the participant demographic information and survey responses, including the median score and graphical illustration of the frequency distribution of response options. Free-text responses in the questionnaires were analyzed using content analysis and collated numerically through frequency data. We used Microsoft Excel for data analyses.

### External Validation

The Delphi results and a final draft of the CC framework for PBT were disseminated and underwent member checking by both consumer and professional stakeholders during annual scientific meetings of COSA, the peak national body for clinical oncology in Australia, and COGNO, the multidisciplinary neuro-oncology clinical trials group, in 2024.^25,26^

## Results

### Multidisciplinary Expert Panel Members

In total, 40 participants (34 HCPs/researchers and 6 consumers) completed the first round of the Delphi survey, and 32 (26 HCPs/researchers and 6 consumers) the second round. Participants who completed the first round were mostly female (*n* = 35, 88%), based in tertiary cancer centers, working in metropolitan areas, with a mean of 8 years (range < 1–25) of experience in neuro-oncology care, research, or support/advocacy (see Table 2). Twenty HCPs (59%) reported a mean of 9 years (range 2–15) of experience overseeing brain tumor CC. Among HCPs, a range of specialties were reported including oncology (*n* = 6, 18%), nursing (*n* = 7, 21%), palliative care (*n* = 1, 3%), diverse allied health (social work, radiation therapy, physiology, psychology; *n* = 12, 36%), and cancer CC (*n* = 4, 12%). Of the 6 consumers, 5 reported a lived experience of PBT.

**Table 2.** Phase 2, Round 1 Delphi Participant Demographics

	Professional (*n* = 34)		Consumer (*n* = 6)	
	*N*	%	*N*	%
Gender				
Female	29	85	6	100
Male	3	9	0	0
Nonbinary	1	3	0	0
Prefer not to disclose	1	3	0	0
Aboriginal or Torres Strait Islander Origin				
No	34	100	6	100
Language as child				
English	28	82	6	100
Other^a^	4	12	0	0
Missing	2	6	0	0
Language spoken at home				
English	28	82	6	100
Other^b^	6	18	0	0
Country of birth				
Australia	22	65	4	67
Aotearoa New Zealand	3	9	0	0
Other^c^	8	24	2	33
Highest level of education				
Higher degree	24	71	2	33
Bachelor	9	26	2	33
Other^d^	1	3	2	33
State of workplace				
New South Wales	17	50	2	33
Queensland	5	15	2	33
Victoria	6	18	2	33
Western Australia	3	9	0	0
Australian Capital Territory	1	3	0	0
South Australia	1	3	0	0
Tasmania	1	3	0	0
Aotearoa New Zealand	0	0	0	0
Location of work				
Metropolitan/Major city	29	85	2	33
Regional/Rural	5	15	4	67
Provision of services in regional/rural areas				
Yes	19	56	1 ^e^	17
No	12	35	4	67
Other	2^f^	6	1 ^g^	17
Lived experience (consumer only)				
Yes			5	83
No			1 ^h^	17
Profession (professional only)				
Social worker	7	21		
Researcher	4	12		
Medical oncologist	4	12		
Clinical nurse consultant	5	15		
Radiation oncologist	2	6		
Radiation therapist	2	6		
Cancer care coordinator	4	12		
Oncology nurse	2	6		
Exercise physiologist	1	3		
Palliative care physician	1	3		
Psychologist	1	3		
Occupational therapist	1	3		
Clinical setting (professional only)				
Tertiary cancer center	22	65		
District/local hospital—Inpatient	4	12		
District/local hospital—Outpatient	10	29		
Private hospital	3	9		
Private cancer treatment center—Outpatient	2	6		
Other ^i^	2	6		
Brain tumor experience/expertise ^j^				
High grade	31	91	4	67
Lower grade	23	68	1	17
Benign	12	35	1	17
Metastatic tumor	20	59	0	0
Did not specify	1	3	1	17
	Mean (range)		Mean (range)	
Years of experience in neuro-oncology care, research, or support/advocacy (*n* = 40)	8.26 (< 1–25)		8.83 (2–15)	
Years of clinical experience, if applicable (*n* = 22)	17 (4–43)		N/A	
Years of experience in care coordination/case management/patient navigation, if applicable (*n* = 20)	9 (2–15)		N/A	

Abbreviations: N/A = Not applicable.

^a,b^Other language as child: Indonesian, Arabic, Tamil, and Polish.

^c^Other country of birth: China, Belgium, Indonesia, England, Lebanon, Sri Lanka, and Poland.

^d^Other levels of education: vocational training, high school.

^e^Both regional and metro.

^f^Phone consults and rural patients visiting center to receive radiation therapy.

^g^Brain cancer charity providing psychological support.

^h^One consumer had no lived experience and worked as a community support worker.

^i^Other clinical settings: University, Tertiary acute hospital.

^j^Multiple selection allowed.

### Phase 1: Item Generation Results

A total of 140 items were identified and organized across 4 domains: (1) Definitions of Coordination of Care (1 item); (2) Objectives of CC (12 items); (3) Components of CC (79 items); and (4) Indicators (48 items). Items in components of CC were grouped into communication, assessment, support, referral, information, tools that facilitate CC, and carer (recognition, assessment, and support). Indicators of CC included items related to person-centered indicators (patient/carer or HCP perspective of care and CC quality and/or outcomes) and performance indicators of governance (eg, healthcare cost, healthcare utilization).

### Phase 2: Consensus Results

**Figure 1.**
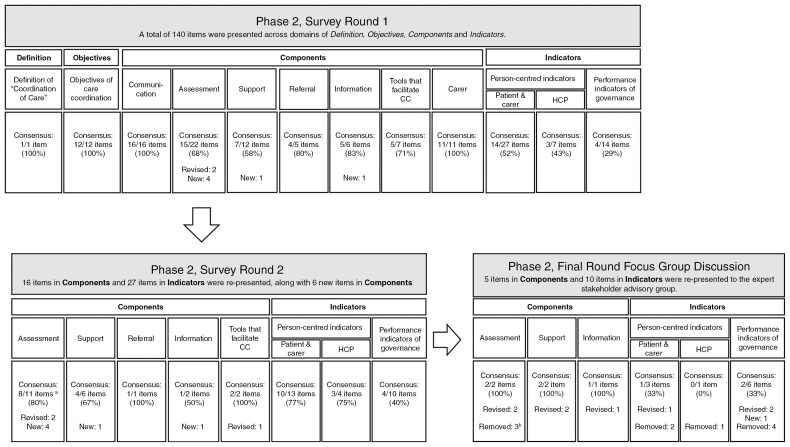
*Phase 2, Delphi Consensus Process.*  ^a^One item was not re-rated as it was replaced with two new items. Total of 10 items were rated. % calculated out of 10. ^b^Three items were removed and replaced with a new item that gained consensus from the previous round. CC, care coordination; HCP, healthcare professional.

Phase 2 consisted of a 2-round Delphi survey and a final focus group discussion among the expert stakeholder advisory group. Figure 1 illustrates the Delphi consensus process and the number of items across domains of CC presented in each round.

#### Round 1.—

All 140 items were presented in the first round of the online survey. Consensus with high agreement (92%) was reached on the single item for **Definition** of *Coordination of Care*. Participants who agreed with this definition commented that it was comprehensive, inclusive, and covered all relevant components required in CC for people with brain tumors and their carers. Agreement was reached for all 12 items in the **Objectives** of CC (range 83%–97% agreement). Items reaching consensus for Definition and Objectives of CC are shown in Box 1.

**Box 1.** Definition and objectives of care coordination in neuro-oncology

**Definition of coordination of care**	
“Coordination of care” is a comprehensive approach to holistic person-centered health care and survivorship support for the duration of brain tumor experience according to the individual patient and family’s needs and preferences, through integration, collaboration, connectedness, and continuity across different service components of multidisciplinary care, and timely delivery of services in an appropriate and complementary manner.	
**Objectives of coordination of care**	
**Item**	**Item description**
Person-centered care	1. To provide appropriate, quality care in response to the individual patient and family’s care needs and preferences
Informed decision-making	2. To clarify clinical care decisions to facilitate informed decision-making for patients and families
Timely access to care	3. To facilitate appropriate, streamlined, and timely service provision to patients and families4. To facilitate timely access to the resources and healthcare system for patients and families
Multidisciplinary care	5. To facilitate access to multidisciplinary care for patients and families
Continuity of care	6. To facilitate the continuity of care over time7. To facilitate the continuity of care between providers
Holistic care	8. To ensure all aspects of the patient and family’s care needs are addressed in a coordinated way, including clinical, social, emotional, practical, and financial implications of disease or illness
Healthcare system navigation	9. To help patients and families navigate the healthcare system
Collaboration and connectedness	10. To facilitate connectivity and communication between hospital and community-based services
High-quality care	11. To improve the quality, safety, and efficiency of overall primary brain tumor care12. To facilitate the objectives and core principles of high-quality care for primary brain tumors. Examples of such objectives or principles (list not exhaustive):- Fostering a compassionate provider–patient relationship- Provision of best evidence-based treatment that is safe and effective- Equity of access to care

Items as presented in Delphi survey in UK/AUS English.

In **Components** of CC, 63/79 items achieved consensus as a core component of CC in the first Delphi round, including all 16 **communication** and all 11 **carer** items. The **carer** items recognize carers as part of PBT care, assessment of carer support needs, and support for carers. Fifteen items (68%) related to **assessment,** 7 items (58%) related to **support**, 4 items (80%) related to **referral**, 5 items (83%) related to **information**, 5 items (71%) related to **tools that facilitate CC** reached consensus in the first round.

Overall, **Indicators** of CC had fewer items achieving consensus (range 29%–52%), particularly for **performance indicators of governance** or healthcare system, where only 4 of the 14 initially met consensus in Round 1. Free-text comments highlighted a lack of valid, objective measures of indicators or potential confounders that may impact the direction/magnitude of indicators of CC.

#### Round 2.—

Forty-three items not reaching consensus were re-presented in Round 2, along with the Round 1 group results and revised items where applicable. We included 6 new items devised from participant comments and suggestions in Round 1. All 5 items listed under **referral** and the 2 revised items for **tools that facilitate CC** reached consensus in this round. After Round 2, 15 items remained unresolved and were discussed in a focus group with the expert stakeholder advisory group (Round 3) for further input based on the aggregate group results, free-text responses, and consumer feedback, and a summary of the research team recommendations. Table 3 presents the finalized list grouped by CC domain.

**Table 3.** Final List of Components of Coordination of Care for Primary Brain Tumor

Components of coordination of care
Communication
Item 1. The whole health service and multidisciplinary care team are responsible for and participate in communication with patients and families and with each other
Item 2. The patient, family, and the multidisciplinary care team know who is involved in the care team and the roles of multidisciplinary healthcare professionals in the patient’s care
Item 3. Pathways for effective communication, a flow of information, and cooperation among the multidisciplinary care team across the hospital, primary care, and community service providers, as well as with patients and families
Item 4. Pathways for effective communication, a flow of information, and cooperation, facilitating transitions between care levels or providers
Item 5. Structured communication pathways—with clearly delineated responsibilities across the multidisciplinary care team
Item 6. Build relationships with the patient, carer, and family through listening and responding
Item 7. At each transition point in care, identify a “key contact” health care professional for patients and families, providing contact information
Item 8. At each transition point in care, establish protocol and triggers for patients and families to contact the identified member of the multidisciplinary care team
Item 9. Understand the immediate impact of primary brain tumor on the patient and family
Item 10. Understand the impact of primary brain tumor over time for the patient and family
Item 11. Understand psychological, interpersonal, or spiritual distress that may accompany primary brain tumor diagnosis for the patient and family.
Item 12. Identify and communicate with the patient and/or family about personality and behavior issues of the patient over time
Item 13. Understand and validate existential distress for the patient and family
Item 14. Identify the need for and facilitate interpreter services where required
Item 15. Identify the need for and facilitate appropriate support when the person’s ability to communicate is impaired due to disability
Item 16. Advocate for the patient and carer within the multidisciplinary care team
Assessment
Item 1. Identify issues and symptoms of the patient and family early and proactively following diagnosis
Item 2. Assess the early post-diagnosis functional and distress levels of the patient and family (establish the baseline)
Item 3. Assess the impact on family dynamics, changing roles of the patient and family members, and social support network following diagnosis
Item 4. Assess whether there is a carer and what role the carer can play/if no carer, whether the patient is isolated
Item 5. Assess the patient self-management capability early: patient self-management knowledge, behaviors, issues, current health and health history, need for coordination and support services
Item 6. Assess the need of assessment of patient’s home environment.
Item 7. Repeated assessment at regular intervals and as needed over time
Item 8. Repeated assessment when there is a change in disease status and/or treatment
Item 9. Clinical assessment according to treatment protocol or standard of care
Item 10. Clinical assessment of symptoms of brain tumors or lesions, side effects of treatment or medication, and clinical signs and symptoms indicating potential tumor changes
Item 11. Consistent data collection of a pre-agreed core dataset
Item 12. Assess the need for support for medication management
Item 13. Assess the need for psychological review and referral
Item 14. Assess behavior and cognitive changes of the patient and the impact on the patient and family
Item 15. Assess the need for neurocognitive review and referral
Item 16. Assess the need for fitness to drive assessment and referral
Item 17. Assess the need for financial support for the patient and family and referral
Item 18. Assess the need for power of attorney
Item 19. Assess the need for enduring guardianship
Item 20. Assess the need for palliative care and referral
Item 21. Assess the need for end-of-life care and referral
Item 22. Assess the need for functional review and referral
Support
Item 1. Provide emotional support to the patient and family
Item 2. Assist and guide the patient and family in individualized treatment planning
Item 3. Assist and guide the patient and family in person-centered survivorship care
Item 4. Assist and guide the patient and family in person-centered end-of-life care
Item 5. Facilitate discussion of enduring power of attorney
Item 6. Facilitate the discussion of guardianship
Item 7. Coordinate appointments with the multidisciplinary care team across the hospital, primary care, and community support service providers
Item 8. Facilitate referral to a psychosocial intervention
Item 9. Assess and facilitate transport needs.
Item 10. Facilitate access to financial navigation and support for patients and families.
Item 11. Coordinate interpreter services
Item 12. Advocate patient empowerment and self-management and provide support required over time when the person’s self-management capability changes
Item 13. Attend appointments where decisions are likely to be made
Referral
Item 1. Assign a healthcare professional to be responsible for overseeing coordination of care and referral pathways
Item 2. Understand referral pathways and provide appropriate referral to patients and families in a timely manner
Item 3. Facilitate a referral to palliative care, allied health services, primary care, or community-based support services when needs are identified
Item 4. Facilitate a referral to healthcare professionals and support services locally available for the patient and family
Item 5. Follow-up with the patient and family after a referral has been made to facilitate access to services where required
Information
Item 1. Provide sufficient and timely information and educational resources to the patient and family as required about the primary brain tumor diagnosis and prognosis
Item 2. Provide sufficient and timely information and educational resources to the patient and family as required about treatment options, clinical trials, side effects, and opportunities for questions
Item 3. Provide sufficient and timely information and educational resources to the patient and family as required about treatment and medication management
Item 4. Provide sufficient and timely information and educational resources to the patient and family as required about symptom management, support, and services
Item 5. Provide information in a format that aids understanding for the patient and family
Item 6. Discuss options of documenting key information from healthcare consultations to aid information recall and retention.
Item 7. Provide information in appropriate languages for patients and families from diverse communities
Tools that facilitate care coordination
Item 1. Participation in multidisciplinary care team meetings
Item 2. Liaising with hospitals, allied health, and/or community-based services
Item 3. Access to telehealth if preferred and clinically safe
Item 4. Timely follow‐up on treatment and symptom management
Item 5. Identifying and connecting with multidisciplinary care services in the patient’s local area
Item 6. Access to brain tumor-related continuing professional development
Item 7. Documentation of patient contacts, information provided to the patient, and support services referred or provided to the patient
Carer recognition, assessment, and support
Recognition
Item 1. Understand the impact of primary brain tumor diagnosis for carers and carer’s own right to appropriate support
Item 2. Articulate the importance of the carer in patient care
Item 3. Recognize the practical support carers provide for patient care
Item 4. Share information and educational resources with carers
Item 5. Recognize the carer’s role as a “navigator” for the patient
Assessment
Item 6. Assess the support needs of carers
Item 7. Screen caregivers for emotional distress throughout the disease trajectory
Support
Item 8. Facilitate access to caregiver respite, emotional support, and referrals to psychological services
Item 9. Identify appropriate support for carers as part of the patient care
Item 10. Facilitate access to financial, legal, or administrative support for carers
Item 11. Provide carers with access to bereavement support

One **assessment** item related to legal support (Item 20) was not re-rated and was instead replaced with 2 new items (Item 25: Assess the need for power of attorney; Item 26: Assess the need for enduring guardianship) to provide clarification. Two additional **assessment** items were also rated (NEW Item 7; Item 8). The expert stakeholder advisory group agreed with removing 3 items related to the need for repeated assessments, given that new Item 7 provided a more holistic approach and covered all aspects reported in the 3 initial items (REMOVED Items 7–9).

Two **assessment** items (Items 6 and 11) did not reach consensus in Round 2. The low agreement for Item 6 (34%) was due to the questioned feasibility of conducting home assessments. The expert stakeholder advisory group agreed with participant comments on this, also noting coordinators collaborate with other allied HCPs (eg, occupational therapist or physiotherapist) to conduct these assessments. The expert group decided to retain Item 6 with revised wording to “assess the need for” home assessments. The expert stakeholder advisory group agreed to retain Item 11 given approaching consensus agreement (75%) but with revised wording of “pre-agreed” dataset to encourage consistent data collection.

Two **support** items (Item 9 coordinate transport; Item 10 coordinate access to funding) did not reach consensus in Round 2. Given Item 10 was just below consensus (76%), the expert stakeholder advisory group retained this item with revised wording (ie, Facilitate access to financial navigation and support for patients and families) given participants’ comments on lack of clarity about the recipient of funding. Although Item 9 did not reach adequate consensus, the expert stakeholder advisory group noted a clear need for this component among patients and retained the item with revised wording, highlighting that CC would *assess and facilitate* transport options for patients rather than coordinate transport.

The single **information** item that did not reach consensus (Item 6: Enable audio-recording of healthcare consultations to aid information recall/retention) was re-presented but did not achieve consensus in Round 2. Reasons for low agreement included provider comfort with audio-recording and hospital policy. However, acknowledging the importance of documentation of key information, the expert stakeholder advisory group suggested rewording the item to ensure that there was a discussion of different options to document key information to aid with information recall and retention. Also, based on participant feedback on information needs of individuals of culturally and linguistically diverse backgrounds, an additional item (Item 7) was presented and achieved consensus.

Overall, 17/27 re-presented items under **Indicators** achieved consensus and were retained by end of Round 2 (see Supplementary Table 2 for full list of indicators with agreement rating), including 10/13 items (77%) for **patient and carer perception**, 3/4 items (75%) for **HCPs’ perception**, and 4/10 items (40%) for **performance indicators of governance**. The expert stakeholder advisory group finalized inclusion, revision, or exclusion of items based on the agreement level of the item in Round 2, discussion of factors other than CC (eg, availability of carer/family members) that could impact a given indicator/outcome (eg, treatment protocol adherence), direct relevance of the indicator to CC, and perceived feasibility of measuring the outcome (eg, cost and time). An additional item related to caseloads (ie, number of patients per full-time equivalent care coordinator) was suggested by the expert stakeholder advisory group, considering the feasibility of measuring the caseload ratio compared to costs (see Table 4 for the final list of indicators).

**Table 4.** Final List of Indicators of Coordination of Care for Primary Brain Tumor

Indicators of coordination of care
Person-centered indicators
The patient or carer’s perception of care and coordination in the following domains:
Item 1. Satisfaction with information
Item 2. Knowledge about treatment and next steps in care
Item 3. Shared decision-making & care planning
Item 4. Understanding the roles of healthcare professionals in the care team
Item 5. Communication with healthcare professionals
Item 6. Emotional support/empathic responses from healthcare professionals
Item 7. Healthcare professionals are aware of patient history and progress (not needing to repeat)
Item 8. Confidence in healthcare professionals
Item 9. Support of the care team in managing the effects of primary brain tumor and treatment
Item 10. Availability of the care team members to provide information when needed
Item 11. Access to care
Item 12. Access to support and services as required
Item 13. Getting timely appointments, care, and information
Item 14. Health-related quality-of-life outcomes
Item 15. Level of unmet needs
Item 16. Carer’s preparedness to care
Item 17. Level of patient self-efficacy.
Item 18. Level of emotional distress
Item 19. Social and functional (re)engagement following treatment
Item 20. Involvement of family members and friends
Item 21. Carer post-bereavement adjustment and satisfaction with the patient’s end-of-life care and dying processes
Item 22. Satisfaction with follow-up and monitoring
Item 23. Patients’ rating of overall cancer care
Item 24. Patients’ rating of support with practical arrangements
Item 25. Overall rating of coordination
The healthcare professional’s perception of coordination of care in the following domains:
Item 1. Toxicity or indicators of toxicity from treatment
Item 2. Test results are ready prior to consultation
Item 3. Cancer care team’s use of information to coordinate patient care
Item 4. Perceived level of communication efficiency
Item 5. Perceived level of information exchange efficiency
Item 6. Knowledge of members’ roles in the multidisciplinary care team and modes of communication with each member
Performance indicators of governance
Item 1. Healthcare utilization prevented due to coordination activities
Item 2. Healthcare utilization as a result of coordination activities
Item 3. Number of appropriate health care and community-based services offered to the patient and family
Item 4. Number of calls/visits to the emergency department for non-life-threatening health concerns.
Item 5. Availability of a healthcare professional responsible for coordination of care
Item 6. Access for the patient to specialists
Item 7. Assessment for eligibility for clinical trials offered to patients and/or carers
Item 8. Time spent on patient interactions
Item 9. Cost of care: costs of information exchange and time investments by healthcare professionals
Item 10. Lead time (time to referral, time to treatment, and total time)
Item 11. Patient needs are considered in multidisciplinary team care planning when documented and/or reported
Item 12. Financial cost for the patient and family
Item 13. Number of patient caseload per full-time equivalent care coordinator

In our secondary analysis, we mapped the indicators to the objectives proposed in our framework, while also identifying relevant principles of the Optimal Care Pathway for High-Grade Glioma^7^ (Supplementary Table 3).

## Discussion

Using the Delphi process, experts and stakeholders in PBT care, support, and advocacy reached consensus on the definition and 12 specific objectives of CC for PBT in AANZ. Both the definition and objectives expand the current knowledge of CC, shifting the focus from patient navigation (eg, directing patients to specialists and scheduling appointments) and access to health care,^7^ to an *approach to holistic person-centered health care and survivorship support for the duration of the brain tumor experience* (Box 1). We also identified a comprehensive list of components of optimal CC and explored a wide range of indicators of quality CC, totaling 136 items that gained consensus through Delphi survey and focus group rounds. The final list included 7 new items and 11 revised items from this iterative process. This framework is the first to guide models of CC that reflect a diverse range of care and support needs and unique treatment requirements for people with PBT.

We focused on developing an optimal CC framework, identifying all components broadly applicable to care processes in all PBT types and across the care continuum. The framework acknowledges the diversity of PBT experiences and care trajectories for PBT subtypes and individual patients’ prognoses.^14,27^ Defining minimum standards for CC with a small set of essential components lacks the flexibility and subtlety that enable a person-centered, holistic approach in brain cancer CC. However, the main challenge for proposing the optimal CC framework is that it may become quality CC *in principle*, but raises questions about the feasibility of addressing most, if not all, components. Current knowledge and practice of CC also rely heavily on a single HCP-led model,^13^ whereas feedback from the multidisciplinary panel members in our Delphi study queried if the framework is achievable within the scope of a care coordinator’s role and skills. Therefore, it is important to clarify that the current framework does not replace a list of duties and responsibilities for a single HCP designated to CC; instead, it serves to increase the awareness of what CC could entail for PBT care and ultimately aid the development and implementation of a CC model suitable for individual service providers.

Compared to components of CC, quality indicators had lower levels of agreement by the multidisciplinary panel members. In particular, we found varying views on performance indicators of governance (or the system of healthcare) in relation to CC, such as healthcare cost and duration of hospital admission. Although these indicators have been used as outcomes of coordinated or integrated care in previous research,^6,8^ many confounders or institutional barriers can impact the direction or magnitude of change in outcome or the feasibility of accurately and systematically assessing outcomes. For instance, healthcare costs may increase as a result of coordination of care when patients access appropriate services for symptoms otherwise unaddressed or inadequately self-managed,^28^ whereas costs may decrease as a result of coordinated care within and across services by avoiding duplication of care or unplanned hospital visits.^7,29^ Singer et al.^30^ proposed measures of CC should assess if the objectives are met for relevant coordination dimensions in relation to the following: coordination within care team; coordination across care teams; coordination between care teams and community resources; continuous familiarity with patient over time; continuous proactive and responsive action between visits; patient-centered; and shared responsibility.^30^ Yet, specific indicators remain preliminary and further empirical evidence is needed to examine their use in benchmarking care delivery and quality.

Importantly, the indicators in our framework provide substantially greater granularity to guide supportive care practice than the principles outlined in the Optimal Care Pathway for High-Grade Glioma, which are general and aspirational in nature. Of note, while we propose several novel indicators, our list of indicators is not entirely brain tumor-specific nor exhaustive, and only indirectly reflects the objective related to facilitating collaboration and connectedness between hospitals and community-based services. The current list does not aim to present the direction of changes in the listed indicators, rather to highlight a diverse range of outcomes warranting further research and clinical consideration when developing and evaluating a CC model or its standards.

### Clinical Implications

This comprehensive framework allows flexibility in tailoring a CC model to varied service contexts and resources in AANZ, thus facilitating its implementation into routine care. In addition, due to the diversity of experiences within PBT, such as tumor subtypes and its rarity, a range of factors can influence modes and components of coordination. This has been similarly raised as issues for developing models of CC in rare chronic health conditions, emphasizing need for flexible, hybrid models of centralized and local care to improve CC.^31^ Selection of components can be adapted for individual patient/family circumstances while maintaining awareness of other components and applying them fluidly as the patient and family’s care and support needs change over time.

In the proposed framework of CC for PBT, **communication** (16 items), **assessment** (22 items), and **support** (13 items) were among the domains that constitute key processes of care and CC for this population. It is important to remain aware of potential issues outlined in **assessment** that may emerge over time and across contexts while regularly assessing the evolving needs of patients and families. This includes balancing the need for screening and comprehensive assessment to facilitate access to specialist assessments by relevant clinicians, allied health professionals, or community support services. Appropriate support and planning for anticipated future care needs should accompany assessment, so HCPs remain vigilant and help individuals with PBT and their carers anticipate and address challenges before a crisis point. As this frequently involves sensitive topics for conversation such as prognosis, palliative and end-of-life care, and legal and financial implications, empathic and person-centered communication is a key component of CC that not only recognizes the informational needs and streamlined communication channels between all personnel involved in patient care, but also the importance of understanding the impact of PBT and communicating this understanding with people with PBT and their families and within the care team.^32^

The high burden of PBT means that many people with PBT often rely on carers or family members for practical and emotional support. Carers of patients diagnosed with PBT have higher levels of carer burden compared to carers of other cancers.^33^ In recognition of this burden and unmet need for carers of people with PBT, all 11 carer-specific items included in our framework achieved high levels of agreement in the first round of the Delphi survey. This highlights the importance of carer involvement and support for carers in a model of CC for PBT.

### Limitations

The Delphi process had limitations, including the sample selection, generalizability, and survey fatigue. While the multidisciplinary panel members represented a wide range of healthcare professions with extensive clinical and neuro-oncology experience and consumer representation, the consensus definition and framework of CC is based on existing knowledge, clinical practice, and resources specific to the AANZ context, although items may be generalizable to other well resourced countries given shared treatment regimens for PBT (eg, NICE pathways, EORTC-26981-22981 study). Hence, the consensus and CC framework may evolve over time and require ongoing discussion and member checking processes to reflect relevant changes necessary for the CC framework. There was unequal representation of professional and consumer stakeholders, and we were unable to determine differences in priorities across these groups, given that this Delphi study aimed to achieve consensus. Future studies can explore the rank/priority of aspects of CC valued by different stakeholder groups to further inform clinical implications of CC models in cost-constrained healthcare settings. Another limitation was the under-representation of participants from rural/regional regions, which impacted on consensus for transport-related items. Input from carers and consumers with culturally and linguistically diverse backgrounds was also lacking. A sample size calculation for statistical power was not applicable, as our data analysis involved the use of descriptive statistics. There is high heterogeneity in the Delphi methodology to guide sample size, and the quality of the Delphi findings depends on the processes related to anonymity, iteration, controlled feedback, and statistical stability of consensus.^34^ We were unable to determine the overall response rate due to overlaps in membership across different peak bodies approached and snowballing recruitment. Our sample consisted of HCPs who had extensive experience of CC in the neuro-oncology context, and this level of knowledge and expertise in CC cannot be assumed for HCPs not directly involved in CC, or in the benign PBT or neurosurgery context. This is not sampling bias, as “multidisciplinary panel members” are recruited as expert panelists for a Delphi study, rather than achieving population representation. Similarly, we purposively recruited consumer stakeholders with reported experience in supporting/advocating for people with PBT in organizations to bring input and perspective from observing a wide range of patient/carer experiences of PBT (eg, community care coordinators or case managers). This helped provide insight into gaps and components relevant to delivering coordinated care in community settings and patients’ local environments. However, most consumer stakeholders were female and had lived experience as survivors. Consumer stakeholders of the expert stakeholder advisory group provided written feedback to inform the final decision on item inclusion/exclusion, but did not participate in the final focus group discussion in person to protect their privacy and for ethical considerations.

### Future Directions

Future studies should investigate the clinical utility of the proposed CC framework and assess how a model of CC can be designed, implemented, and evaluated in a clinical setting as guided by implementation frameworks, such as Consolidated Framework for Implementation Research.^35^ In addition, the framework may provide a useful foundation for developing tools to aid CC, such as a checklist, position description of an HCP for the role in CC, or to build common knowledge/standards among care team members that are relevant and appropriate to their practice setting and resources.

In conclusion, our study presents a novel framework of CC for people with PBT derived through a multistage Delphi consensus process. This framework includes a total of 136 items, encompassing the definition and objectives of CC, as well as a comprehensive list of components and indicators to guide the development and evaluation of an optimal CC model in neuro-oncology. Further research is recommended to explore the clinical utility and translation of this framework into clinical practice, and further investigate the direction and magnitude of change in indicators to benchmark quality CC in PBT.

## Supplementary material

Supplementary material is available online at *Neuro-Oncology Practice* (https://academic.oup.com/nop/).

npaf082_Supplementary_Figures_1_Tables_2-3

## Data Availability

The data will be made available upon reasonable request in line with the ethical guidelines and conditions outlined in the participant consent.

## References

[1] Ford E, Catt S, Chalmers A, Fallowfield L. Systematic review of supportive care needs in patients with primary malignant brain tumors. Neuro Oncol. 2012;14(4):392–404. 22307475 10.1093/neuonc/nor229 PMC3309849

[2] Goebel S, Stark AM, Kaup L, von Harscher M, Mehdorn HM. Distress in patients with newly diagnosed brain tumours. Psychooncology. 2011;20(6):623–630. 21449043 10.1002/pon.1958

[3] Trad W, Koh ES, Daher M, et al Screening for psychological distress in adult primary brain tumor patients and caregivers: considerations for cancer care coordination. Front Oncol. 2015;5:203. 26442215 10.3389/fonc.2015.00203 PMC4585197

[4] Halkett GKB, Lobb EA, Rogers MM, et al Predictors of distress and poorer quality of life in high grade glioma patients. Patient Educ Couns. 2015;98(4):525–532. 25638306 10.1016/j.pec.2015.01.002

[5] Renovanz M, Hechtner M, Janko M, et al Factors associated with supportive care needs in glioma patients in the neuro-oncological outpatient setting. J Neurooncol. 2017;133(3):653–662. 28527007 10.1007/s11060-017-2484-y

[6] Schultz EM, McDonald KM. What is care coordination? Int J Care Coord. 2014;17(1-2):5–24.

[7] Cancer Council Victoria and Department of Health Victoria. Optimal Care Pathway for People With High-Grade Glioma. Melbourne: Cancer Council Victoria; 2021.

[8] Gorin SS, Haggstrom D, Han PKJ, et al Cancer care coordination: a systematic review and meta-analysis of over 30 years of empirical studies. Ann Behav Med. 2017;51(4):532–546. 28685390 10.1007/s12160-017-9876-2

[9] Freeman HP, Rodriguez RL. History and principles of patient navigation. Cancer. 2011;117(S15):3537–3540. 21780088 10.1002/cncr.26262 PMC4557777

[10] Clinical Oncology Society of Australia. Cancer Care Coordinator Position Statement. 2015. Sydney, NSW, Australia: Clinical Oncology Society of Australia.

[11] Miller E. Neuro-oncology nurse navigation: developing the role for a unique patient population. Clin J Oncol Nurs. 2018;22(3):347–349. 29781470 10.1188/18.CJON.347-349

[12] Halkett GKB, Berg MN, Daudu D, et al Supportive care of patients diagnosed with high grade glioma and their carers in Australia. J Neurooncol. 2022;157(3):475–485. 35397081 10.1007/s11060-022-03991-z PMC8994178

[13] Jeon MS, Banks H, He S, et al; for the BRAINS Investigator Group. Identifying components of care coordination for primary brain tumor: a scoping review. Neurooncol. Pract.. 2025;12(3):357–375. 40487588 10.1093/nop/npaf003 PMC12137213

[14] Philip J, Collins A, Brand C, et al A proposed framework of supportive and palliative care for people with high-grade glioma. Neuro Oncol. 2018;20(3):391–399. 29016886 10.1093/neuonc/nox140 PMC5817948

[15] Osoba D, Aaronson NK, Muller M, et al Effect of neurological dysfunction on health-related quality of life in patients with high-grade glioma. J Neurooncol. 1997;34(3):263–278. 9258818 10.1023/a:1005790632126

[16] Bailey A, Trad W, Kastelan M, Lamont S. Australian experience of neuro-oncology care coordination: a conversation. Clin J Oncol Nurs. 2015;19(5):610–614. 26414579 10.1188/15.CJON.610-614

[17] Iqbal S, Pipon-Young L. The Delphi method. Psychologist. 2009;22(7):598–600.

[18] Halkett GKB, Breen LJ, Berg M, et al Determining the research priorities for adult primary brain tumours in Australia and New Zealand: a Delphi STudy with consumers, health professionals, and researchers. Curr Oncol. 2022;29(12):9928–9955. 36547195 10.3390/curroncol29120781 PMC9777470

[19] Tyler N, Planner C, Byrne M, et al Developing best practice guidance for discharge planning using the RAND/UCLA appropriateness method. Front Psychiatry. 2021;12:789418. 34925112 10.3389/fpsyt.2021.789418 PMC8680088

[20] Jünger S, Payne SA, Brine J, Radbruch L, Brearley SG. Guidance on Conducting and REporting DElphi Studies (CREDES) in palliative care: recommendations based on a methodological systematic review. Palliat Med. 2017;31(8):684–706. 28190381 10.1177/0269216317690685

[21] Harris PA, Taylor R, Thielke R, et al Research electronic data capture (REDCap)—a metadata-driven methodology and workflow process for providing translational research informatics support. J Biomed Inform. 2009;42(2):377–381. 18929686 10.1016/j.jbi.2008.08.010 PMC2700030

[22] Barrett D, Heale R. What are Delphi studies? Evid Based Nurs. 2020;23(3):68–69. 32430290 10.1136/ebnurs-2020-103303

[23] Fitch K, Bernstein SJ, Aguilar MD, et al The Rand / UCLA Appropriateness Method User’ S manual. Santa Monica: RAND; 2001.

[24] Leyenaar MS, Strum RP, Batt AM, et al Examining consensus for a standardised patient assessment in community paramedicine home visits: a RAND/UCLA-modified Delphi Study. BMJ Open. 2019;9(10):e031956. 10.1136/bmjopen-2019-031956 PMC6797257 31594901

[25] Jeon MS, He S, Shaw J, et al What constitutes optimal care coordination for primary brain tumours and how do we assess it in Australia: a Delphi consensus study. Asia-Pacific J Clin Oncol. 2024;20(S3):91–92. 10.1093/nop/npaf082 PMC12965651 41798133

[26] Jeon MS, He S, Shaw J, et al What constitutes optimal care coordination for primary brain tumours and how do we assess it in Australia: a Delphi consensus study. Paper presented at: 16 th COGNO Annual Scientific Meeting. Precision Targets: Personalised Care in Neuro-Oncology; October 13–15, 2024; Melbourne, Australia.

[27] Philip J, Collins A, Brand CA, et al Health care professionals’ perspectives of living and dying with primary malignant glioma: implications for a unique cancer trajectory. Palliat Support Care. 2015;13(6):1519–1527. 24138726 10.1017/S1478951513000576

[28] Ålykkja A, Ruud E, Larsen MH, Vatne TM, Lie HC. Available, but not always accessible: a nationwide, qualitative study of multidisciplinary healthcare providers’ experiences with follow-up care after paediatric brain tumour. Eur J Cancer Care (Engl). 2021;30(2):e13375. 33236388 10.1111/ecc.13375

[29] Hong M, Leigh L, Ballinger C, et al The impact of brain cancer care coordinators on healthcare utilization and outcomes in patients with glioblastoma. Neurooncol. Pract.. 2024;11(5):575–582. 39279777 10.1093/nop/npae030 PMC11398931

[30] Singer SJ, Burgers J, Friedberg M, et al Defining and measuring integrated patient care: promoting the next frontier in health care delivery. Med Care Res Rev. 2011;68(1):112–127. 20555018 10.1177/1077558710371485

[31] Walton H, Simpson A, Ramsay AIG, et al Development of models of care coordination for rare conditions: a qualitative study. Orphanet J Rare Dis. 2022;17(1):49. 35164822 10.1186/s13023-022-02190-3 PMC8843018

[32] Legge DM, Jeon M, Banks H, et al P22.04.B balancing communication challenges in neuro-oncology: a cross-sectional interview study with neuro-oncology healthcare professionals. Neuro Oncol. 2024;26(Suppl_5):v123–v123.

[33] Aoun SM, Deas K, Howting D, Lee G. Correction: exploring the support needs of family caregivers of patients with brain cancer using the CSNAT: a comparative study with other cancer groups. PLoS One. 2016;11(1):e0148074. 26799832 10.1371/journal.pone.0148074 PMC4723223

[34] de Villiers MR, de Villiers PJ, Kent AP. The Delphi technique in health sciences education research. Med Teach. 2005;27(7):639–643. 16332558 10.1080/13611260500069947

[35] Damschroder LJ, Reardon CM, Widerquist MAO, Lowery J. The updated consolidated framework for implementation research based on user feedback. Implement Sci. 2022;17(1):75. 36309746 10.1186/s13012-022-01245-0 PMC9617234

