# Supplementary material for: What constitutes optimal care coordination for primary brain tumors and how do we assess it in Australia and Aotearoa New Zealand? A Delphi consensus study
Source: Neurooncol Pract. 2025 Aug 4;13(1):162–77. doi: 10.1093/nop/npaf082 (PMC12965651; doi:10.1093/nop/npaf082)
Supplement: npaf082_Supplementary_Figures_1_Tables_2-3 [file npaf082_supplementary_figures_1_tables_2-3.docx]

**Supplementary Figure 1.**

*Delphi Study Flow*

**Supplementary Materials.**

**Definition of Indicators**

Indicators refer to constructs or outcomes potentially used to assess components, features, processes/delivery or outcomes of models of CC in terms of meeting the objectives of CC (e.g., facilitating timely, high-quality brain tumour care). Indicators generated from Phase 1 included person-centred outcomes (i.e., perception of patient/carer and of HCPs towards coordination and delivery of care) and performance indicators of governance (i.e., quality of performance and processes of the system in coordinating care).

**Supplementary Table 1.**

*Overall Ratings for Components of Care Coordination.*

| COMPONENTS OF COORDINATION OF CARE | Level of Consensus (%) | Median Rating (range) | Consensus Round |
| --- | --- | --- | --- |
| Communication |  |  |  |
| Item 1. The whole health service and multi-disciplinary care team is responsible for and participates in communication with patients and families and with each other | 89 | 4 (2-5) | 1 |
| Item 2. The patient, family, and the multi-disciplinary care team know who is involved in the care team and the roles of multi-disciplinary healthcare professionals in the patient’s care | 97 | 4 (3-5) | 1 |
| Item 3. Pathways for effective communication, a flow of information, and cooperation amongst the multi-disciplinary care team across the hospital, primary care, and community service providers, as well as with patients and families | 97 | 4 (4-5) | 1 |
| Item 4. Pathways for effective communication, a flow of information, and cooperation facilitating transitions between care levels or providers | 91 | 4 (3-5) | 1 |
| Item 5. Structured communication pathways – with clearly delineated responsibilities across the multidisciplinary care team | 80 | 4 (3-5) | 1 |
| Item 6. Build relationships with the patient, carer and family through listening and responding | 100 | 5 (4-5) | 1 |
| Item 7. At each transition point in care, identify a “key contact” health care professional for patients and families, providing contact information | 89 | 4 (3-5) | 1 |
| Item 8. At each transition point in care, establish protocol and triggers for patients and families to contact the identified member of the multi-disciplinary care team | 83 | 4 (3-5) | 1 |
| Item 9. Understand the immediate impact of primary brain tumour for the patient and family | 88 | 4 (3-5) | 1 |
| Item 10. Understand the impact of primary brain tumour over time for the patient and family | 91 | 4 (3-5) | 1 |
| Item 11. Understand psychological, interpersonal, or spiritual distress that may accompany primary brain tumour diagnosis for the patient and family. | 94 | 4 (3-5) | 1 |
| Item 12. Identify and communicate with the patient and/or family about personality and behaviour issues of the patient over time | 97 | 4 (3-5) | 1 |
| Item 13. Understand and validate existential distress for the patient and family | 97 | 4 (3-5) | 1 |
| Item 14. Identify the need for and facilitate interpreter services where required | 97 | 4 (3-5) | 1 |
| Item 15. Identify the need for and facilitate appropriate support when the person’s ability to communicate is impaired due to disability | 100 | 4 (4-5) | 1 |
| Item 16. Advocate for the patient and carer within the multi-disciplinary care team | 94 | 5 (3-5) | 1 |
| Assessment |  |  |  |
| Item 1. Identify issues and symptoms of the patient and family early and proactively following diagnosis | 80 | 4 (3-5) | 1 |
| Item 2. Assess the early post-diagnosis functional and distress levels of the patient and family (establish the baseline) | 87 | 4 (3-5) | 1 |
| Item 3. Assess the impact on family dynamics, changing roles of the patient and family members, and social support network following diagnosis | 93 | 4 (3-5) | 1 |
| Item 4. Assess whether there is a carer and what role the carer can play/ if no carer, whether the patient is isolated | 100 | 5 (4-5) | 1 |
| Item 5. Assess the patient self-management capability early: patient self-management knowledge, behaviours, issues, current health and health history, need for coordination and support services | 93 | 5 (3-5) | 1 |
| [REVISED] Item 6. Assess the need for assessment of patient’s home environment. | N/A (34) | N/A (3(1-5)) | 3 (2) |
| [NEW] Item 7. Repeated assessment at regular intervals and as needed over time | 97 | 4 (3-5) | 2 |
| [REMOVED]Item 7. Repeated assessment at regular interval | 83 | 4 (3-5) | 2 |
| [REMOVED]Item 8. Repeated assessment as needed | 93 | 4 (3-5) | 2 |
| [REMOVED] Item 9. Repeated assessment over time | 93 | 4 (2-5) | 2 |
| [NEW] Item 8. Repeated assessment when there is a change in disease status and/or treatment | 97 | 5 (3-5) | 1 |
| [REVISED] Item 9. Clinical assessment according to treatment protocol or standard of care | 93 | 4 (3-5) | 2 |
| Item 10. Clinical assessment of symptoms of brain tumours or lesions, side-effects of treatment or medication, and clinical signs and symptoms indicating potential tumour changes | 83 | 4 (3-5) | 1 |
| [REVISED] Item 11. Consistent data collection of a pre-agreed core dataset | N/A (76) | N/A (4 (3-5)) | 3 (2) |
| Item 12. Assess the need for support for medication management | 82 | 4 (2-5) | 1 |
| Item 13. Assess the need for psychological review and referral | 97 | 4 (3-5) | 1 |
| Item 14. Assess behaviour and cognitive changes of the patient and the impact on the patient and family | 96 | 5 (3-5) | 1 |
| Item 15. Assess the need for neurocognitive review and referral | 82 | 4 (3-5) | 1 |
| Item 16. Assess the need for fitness to drive assessment and referral | 83 | 4 (3-5) | 1 |
| Item 17. Assess the need for financial support for the patient and family and referral | 97 | 4 (3-5) | 1 |
| [NEW] Item 18. Assess the need for power of attorney | 83 | 4 (2-5) | 2 |
| [NEW] Item 19. Assess the need for enduring guardianship | 83 | 4 (2-5) | 2 |
| [REMOVED] ITEM 20. Assess the need for legal support for the patient and family and referral | N/A (72%) | N/A (4 (2-5)) | 1 |
| Item 20. Assess the need for palliative care and referral | 97 | 5 (3-5) | 1 |
| Item 21. Assess the need for end-of-life care and referral | 93 | 5 (3-5) | 1 |
| [NEW] Item 22. Assess the need for functional review and referral | 97 | 4 (3-5) | 2 |
| Support |  |  |  |
| Item 1. Provide emotional support to the patient and family | 93 | 4 (3-5) | 1 |
| Item 2. Assist and guide the patient and family in individualised treatment planning | 85 | 4 (2-5) | 1 |
| Item 3. Assist and guide the patient and family in person-centred survivorship care | 89 | 4 (3-5) | 1 |
| Item 4. Assist and guide the patient and family in person-centred end-of-life care | 96 | 4 (3-5) | 1 |
| Item 5. Facilitate discussion of enduring power of attorney | 93 | 4 (3-5) | 2 |
| Item 6. Facilitate the discussion of guardianship | 83 | 4 (3-5) | 2 |
| Item 7. Coordinate appointments with the multi-disciplinary care team across the hospital, primary care, and community support service providers | 89 | 5 (1-5) | 1 |
| Item 8. Facilitate referral to a psychosocial intervention | 93 | 4 (3-5) | 1 |
| [REVISED] Item 9. Assess and facilitate transport needs. | N/A (64) | N/A (4(3-5)) | 3 (2) |
| [REVISED] Item 10. Facilitate access to financial navigation and support for patients and families. | N/A (76) | N/A (4(3-5)) | 3 (2) |
| [REVISED] Item 11. Coordinate interpreter services | 83 | 4(3-5) | 2 |
| Item 12. Advocate patient empowerment and self-management and provide support required over time when the person's self-management capability changes | 93 | 4 (3-5) | 1 |
| [NEW] Item 13. Attend appointments where decisions are likely to be made | 86 | 4 (3-5) | 2 |
| Referral |  |  |  |
| Item 1. Assign a healthcare professional to be responsible for overseeing coordination of care and referral pathways | 100 | 4 (4-5) | 2 |
| Item 2. Understand referral pathways and provide appropriate referral to patients and families in a timely manner | 96 | 5 (3-5) | 1 |
| Item 3. Facilitate a referral to palliative care, allied health services, primary care or community-based support services when needs are identified | 96 | 5 (3-5) | 1 |
| Item 4. Facilitate a referral to healthcare professionals and support services locally available for the patient and family | 100 | 4 (4-5) | 1 |
| Item 5. Follow up with the patient and family after a referral has been made to facilitate access to services where required | 93 | 4 (3-5) | 1 |
| Information |  |  |  |
| Item 1. Provide sufficient and timely information and educational resources to the patient and family as required about the primary brain tumour diagnosis and prognosis | 100 | 4 (4-5) | 1 |
| Item 2. Provide sufficient and timely information and educational resources to the patient and family as required about treatment options, clinical trials, and side effects, and opportunities for questions | 89 | 4 (2-5) | 1 |
| Item 3. Provide sufficient and timely information and educational resources to the patient and family as required about treatment and medication management | 96 | 4 (3-5) | 1 |
| Item 4. Provide sufficient and timely information and educational resources to the patient and family as required about symptom management, support and services | 100 | 5 (4-5) | 1 |
| Item 5. Provide information in a format that aids understanding for the patient and family | 100 | 5 (4-5) | 1 |
| [REVISED] Item 6. Discuss options of documenting key information from healthcare consultations to aid information recall and retention. | N/A (52) | N/A (4(2-5)) | 3 (2) |
| [NEW] Item 7. Provide information in appropriate languages for patients and families from diverse communities | 97 | 4 (3-5) | 2 |
| Tools that facilitate care coordination |  |  |  |
| Item 1. Participation in multi-disciplinary care team meetings | 89 | 5 (3-5) | 1 |
| Item 2. Liaising with hospitals, allied health and/or community-based services | 96 | 5 (3-5) | 1 |
| Item 3. Access to telehealth if preferred and clinically safe | 89 | 4 (3-5) | 2 |
| Item 4. Timely follow‐up on treatment and symptom management | 89 | 5 (3-5) | 1 |
| Item 5. Identifying and connecting with multi-disciplinary care services in the patient's local area | 82 | 5 (3-5) | 1 |
| [REVISED] Item 6. Access to brain tumour related continuing professional development | 100 | 4 (4-5) | 2 |
| Item 7. Documentation of patient contacts, information provided to the patient and support services referred or provided to the patient | 86 | 4 (3-5) | 1 |
| Carer |  |  |  |
| *Recognition* |  |  |  |
| Item 1. Understand the impact of primary brain tumour diagnosis for carers and carer’s own right to appropriate support | 96 | 5 (3-5) | 1 |
| Item 2. Articulate the importance of the carer in the patient care | 93 | 4.5 (3-5) | 1 |
| Item 3. Recognise the practical support carers provide for the patient care | 96 | 4.5 (3-5) | 1 |
| Item 4. Share information and educational resources with carers | 100 | 5 (4-5) | 1 |
| Item 5. Recognise the carer’s role as a ‘navigator’ for the patient | 93 | 4 (3-5) | 1 |
| *Assessment* |  |  |  |
| Item 6. Assess the support needs of carers | 100 | 5 (4-5) | 1 |
| Item 7. Screen caregivers for emotional distress throughout the disease trajectory | 96 | 4 (3-5) | 1 |
| *Support* |  |  |  |
| Item 8. Facilitate access to caregiver respite, emotional support, and referrals to psychological services | 100 | 5 (4-5) | 1 |
| Item 9. Identify appropriate support for carers as part of the patient care | 100 | 4.5 (4-5) | 1 |
| Item 10. Facilitate access to financial, legal, or administrative support for carers | 96 | 4 (3-5) | 1 |
| Item 11. Provide carers with access to bereavement support | 96 | 4 (3-5) | 1 |

*Note.* Two revised items and six new items were added to Round 2 online survey following participant feedback from Round 1. Items that did not reach consensus by the end of Round 2 online survey were presented in Round 3 (Focus group) to the Expert Stakeholder Advisory Group. The group made decisions to either remove or revise the wording of items. Carer related items in the Components are listed in Table 2.

There were eight items with revised wording. The original wording for revised items 6, 9 and 11 under Assessments were “Assess the patient's home environment through in-person home visit”, “Repeated assessment over time” and “Consistent data collection of a core dataset to inform high-quality care and care outcomes” respectively. The original wording for Items 9, 10 and 11 under Support were “Coordinate transport”, “Coordinate access to funding”, “Coordinate interpreter services”. The original wording for Item 6 under Information was “Enable audio-recording of healthcare consultations to aid information recall/retention”, and finally Item 6 under Tools that facilitate care coordination was “Continuing professional development”.

**Supplementary Table 2.**

*Overall Rating for Indicators of Quality Care Coordination.*

| **INDICATORS OF COORDINATION OF CARE** | Levels of consensus (%) | Median Rating (range) | Consensus Round |
| --- | --- | --- | --- |
| Person-centred indicators |  |  |  |
| The patient or carer's perception of care and coordination in the following domains: |  |  |  |
| Item 1. Satisfaction with information | 96 | 4 (3-5) | 2 |
| Item 2. Knowledge about treatment and next steps in care | 89 | 4 (3-5) | 1 |
| Item 3. Shared decision-making & care planning | 93 | 4 (3-5) | 1 |
| Item 4. Understanding the roles of healthcare professionals in the care team | 82 | 4 (2-5) | 1 |
| Item 5. Communication with healthcare professionals | 86 | 4 (3-5) | 1 |
| Item 6. Emotional support/empathic responses from healthcare professionals | 82 | 5 (3-5) | 1 |
| Item 7. Healthcare professionals aware of patient history and progress (not needing to repeat) | 100 | 4 (4-5) | 2 |
| Item 8. Confidence in healthcare professionals | 96 | 4 (3-5) | 1 |
| Item 9. Support of the care team in managing the effects of primary brain tumour and treatment | 86 | 4 (3-5) | 1 |
| Item 10. Availability of the care team members to provide information when needed | 100 | 4 (4-5) | 2 |
| [REMOVED] Item 11. Helpful, courteous, and respectful office staff. | N/A (71) ^a^ | N/A (4 (3-5)) | 3 (2) |
| Item 12. Access to care | 92 | 4 (3-5) | 1 |
| Item 13. Access to support and services as required | 96 | 4 (3-5) | 1 |
| Item 14. Getting timely appointments, care, and information | 85 | 4 (3-5) | 1 |
| Item 15. Health-related quality of life outcomes | 93 | 4 (3-5) | 2 |
| Item 16. Level of unmet needs | 81 | 4 (2-5) | 1 |
| Item 17. Carer’s preparedness to care | 100 | 4 (4-5) | 1 |
| **[REVISED] Item 18. Level of patient self-efficacy.** | **N/A (79)** | **N/A (4 (3-5)** | **3 (2)** |
| Item 19. Level of emotional distress | 93 | 4 (3-5) | 2 |
| Item 20. Social and functional (re)engagement following treatment | 96 | 4 (3-5) | 2 |
| Item 21. Involvement of family members and friends | 93 | 4 (3-5) | 2 |
| Item 22. Carer post-bereavement adjustment and satisfaction with the patient's end-of-life care and dying processes | 85 | 4 (3-5) | 1 |
| Item 23. Satisfaction with follow-up and monitoring | 85 | 4 (3-5) | 2 |
| [REMOVED] Item 24. Patients and/or carers' rating of the care team | N/A (68) | N/A (4 (3-5)) | 3 (2) |
| Item 25. Patients’ rating of overall cancer care | 82 | 4 (3-5) | 2 |
| Item 26. Patients' rating of support with practical arrangements | 86 | 4 (3-5) | 2 |
| Item 27. Overall rating of coordination | 81 | 4 (3-5) | 1 |
| The healthcare professional’s perception of coordination of care in the following domains: |  |  |  |
| [REMOVED] Item 1. Treatment protocol adherence of the patient | N/A (69) | N/A (4 (1-5)) | 3 (2) |
| Item 2. Toxicity or indicators of toxicity from treatment | 89 | 4 (1-5) | 2 |
| Item 3. Test results ready prior to consultation | 93 | 4 (3-5) | 2 |
| Item 4. Cancer care team’s use of information to coordinate patient care | 89 | 4 (3-5) | 1 |
| Item 5. Perceived level of communication efficiency | 89 | 4 (3-5) | 1 |
| Item 6. Perceived level of information exchange efficiency | 86 | 4 (3-5) | 1 |
| Item 7. Knowledge of members' roles in the MDT and modes of communication with each member | 89 | 4 (3-5) | 2 |
| Performance indicators of governance |  |  |  |
| [REMOVED] Item 1. Healthcare costs across the whole health system | N/A (63) | N/A (4 (1-5)) | 3 (2) |
| Item 2. Healthcare utilisation prevented due to coordination activities | 96 | 4 (3-5) | 2 |
| Item 3. Healthcare utilisation as a result of coordination activities | 92 | 4 (3-5) | 2 |
| [REMOVED] Item 4. Number of days of hospital admission across different disease phases | N/A (56) | N/A (4 (2-5)) | 3 (2) |
| **[REVISED] Item 5. Number of appropriate healthcare and community-based services offered to the patient and family** | **N/A (78)** | **N/A (4(2-5))** | **3 (2)** |
| **[REVISED] Item 6. Number of calls/visits to the emergency department for non-life threatening health concerns.** | **N/A (74)** | **N/A (4(2-5))** | **3 (2)** |
| Item 7. Availability of a healthcare professional responsible for coordination of care | 89 | 4 (3-5) | 1 |
| Item 8. Access for the patient to specialists | 100 | 4 (4-5) | 2 |
| Item 9. Assessment for eligibility for clinical trials offered to patients and/or carers | 100 | 4 (4-5) | 2 |
| [REMOVED] Item 10. Time spent on patient interactions | N/A (59) | N/A (4(3-5)) | 3 (2) |
| [REMOVED] Item 11. Cost of care: costs of information exchange and time investments by healthcare professionals | N/A (74) | N/A (4(1-5)) | 3 (2) |
| Item 12. Lead time (time to referral, time to treatment and total time) | 81 | 4 (3-5) | 1 |
| Item 13. Patient needs are considered in MDT care planning when documented and/or reported | 89 | 4 (3-5) | 1 |
| Item 14. Financial cost for the patient and family | 86 | 4 (2-5) | 1 |
| **[NEW] Volume/Number of Patient Caseload per FTE care coordinator** | **N/A** | **N/A** | **3** |

^a^ Items marked [REVISED/REMOVED/NEW] did not achieve consensus in Round 1 or 2, hence presented to the Expert group in Round 3. Numbers are the consensus agreement (%) and median score from Round 2.

*Note.* Items that did not reach consensus by the end of Phase 2, Round 2 online survey were presented in Round 3 to the Expert Advisory Group. The group made decisions to either remove or revise the wording of items.

The original wording for Item 5. Number of healthcare and community-based services offered to the patient and family was revised to “Number of appropriate healthcare and community-based services offered to the patient and family”; Item 6. Number of calls/visits to the emergency department was revised to “Number of calls/visits to the emergency department for non-life-threatening health concerns” and Item 18 Patient Self-Management was revised to “Level of Patient Self-Efficacy”.

**Supplementary Table 3.** Mapping of indicators to the objectives of CC and the Principles of Optimal Care Pathway.^12^

| **Objectives** | **INDICATORS OF COORDINATION OF CARE** | **Principles of The Optimal Care Pathway** |
| --- | --- | --- |
| All | PC Item 27. Overall rating of coordination | Care coordination & Multidisciplinary care |
| Continuity of care | PC Item 7. Healthcare professionals aware of patient history and progress (not needing to repeat) |  |
|  | PC Item 23. Satisfaction with follow-up and monitoring |  |
|  | HCP Item 4. Cancer care team’s use of information to coordinate patient care |  |
|  | HCP Item 5. Perceived level of communication efficiency |  |
|  | HCP Item 6. Perceived level of information exchange efficiency |  |
| Healthcare system navigation | G/S Item 2. Healthcare utilisation prevented due to coordination activities |  |
|  | G/S Item 3. Healthcare utilisation as a result of coordination activities |  |
|  | G/S Item 7. Availability of a healthcare professional responsible for coordination of care |  |
|  | G/S Item 6. Number of calls/visits to the emergency department for non-life threatening health concerns. |  |
|  | G/S Item 8. Access for the patient to specialists |  |
|  | G/S Item 15. "Volume/number of patient caseload per FTE care coordinator" |  |
| Timely access to care | PC Item 10. Availability of the care team members to provide information when needed |  |
|  | PC Item 14. Getting timely appointments, care, and information |  |
|  | PC Item 12. Access to care |  |
|  | PC Item 13. Access to support and services as required |  |
|  | G/S Item 12. Lead time (time to referral, time to treatment and total time) |  |
|  | HCP Item 3. Test results ready prior to consultation |  |
| Multi-disciplinary care | PC Item 4. Understanding the roles of healthcare professionals in the care team | (Not covered) |
|  | G/S Item 5. Number of appropriate healthcare and community-based services offered to the patient and family | Supportive care |
| Person-centred care | PC Item 21. Involvement of family members and friends | Patient-centred care |
|  | G/S Item 13. Patient needs are considered in MDT care planning when documented and/or reported |  |
|  | PC Item 1. Satisfaction with information |  |
| Informed decision making | PC Item 3. Shared decision-making & care planning |  |
|  | G/S Item 9. Assessment for eligibility for clinical trials offered to patients and/or carers |  |
| High quality care | PC Item 25. Patients’ rating of overall cancer care |  |
|  | PC Item 5. Communication with healthcare professionals |  |
|  | PC Item 2. Knowledge about treatment and next steps in care |  |
|  | PC Item 8. Confidence in healthcare professionals | Safe and quality care |
|  | HCP Item 2. Toxicity or indicators of toxicity from treatment |  |
|  | HCP Item 7. Knowledge of members' roles in the MDT and modes of communication with each member | Care coordination |
|  | PC Item 9. Support of the care team in managing the effects of primary brain tumour and treatment | Supportive care |
| Holistic care | PC Item 17. Carer’s preparedness to care | (Not covered) |
|  | PC Item 6. Emotional support/empathic responses from healthcare professionals |  |
|  | PC Item 22. Carer post-bereavement adjustment and satisfaction with the patient's end-of-life care and dying processes |  |
|  | PC Item 18. Level of patient self-efficacy. | Patient-centred care |
|  | G/S Item 14. Financial cost for the patient and family |  |
|  | PC Item 15. Health-related quality of life outcomes | Supportive care |
|  | PC Item 16. Level of unmet needs |  |
|  | PC Item 19. Level of emotional distress |  |
|  | PC Item 20. Social and functional (re)engagement following treatment |  |
|  | PC Item 26. Patients' rating of support with practical arrangements |  |

Abbreviations. PC = indicators related to patient and carer perspectives of CC; HCP = indicators related to healthcare professionals’ perception of CC; G/S = Performance indicators of governance or system.
